# Mycological evaluation of frozen meat with special reference to yeasts

**DOI:** 10.14202/vetworld.2023.571-579

**Published:** 2023-03-22

**Authors:** Hams M. A. Mohamed, Sulaiman F. Aljasir, Rofida F. Moftah, Waleed Younis

**Affiliations:** 1 Department of Microbiology, Faculty of Veterinary Medicine, South Valley University, Qena, Egypt; 2 Department of Veterinary Medicine, College of Agriculture and Veterinary Medicine, Qassim University, Buraydah, Saudi Arabia; 3 Department of Food Science and Technology, Faculty of Agriculture, Assiut University, Assiut, Egypt

**Keywords:** antimicrobial resistance, beef, biofilm, enzymatic activities, fungi, meat, yeast

## Abstract

**Background and Aim:**

Fungi can play beneficial and detrimental roles in meat products; however, the diversity and significance of fungi in meat products are poorly understood. This study aimed to isolate and characterize fungal species from frozen beef samples collected from retail stores in the Qena Governorate, Egypt.

**Materials and Methods:**

A total of 70 frozen beef samples were collected from retail stores in Qena, Egypt. All samples were subjected to mycological examination. Fungal colonies were identified using conventional approaches, as well as the VITEK 2 system and DNA sequencing of the internal transcribed spacer region. Analyses of enzymatic activity, biofilm formation ability, and the antimicrobial resistance profiles of the isolated yeasts were also conducted.

**Results:**

Molds and yeasts were isolated from 40% and 60% of meat samples, respectively. Mold isolates were dominated by *Aspergillus*, *Penicillium*, and *Cladosporium* spp., whereas yeast isolates were identified as *Candida* *albicans*, *Candida parapsilosis*, *Yarrowia lipolytica*, *Saccharomyces cerevisiae*, and *Rhodotorula mucilaginosa*. Compared to other yeast species, the highest production of lipase and protease was observed in *Candida* species. The strongest ability to form biofilms was observed in *Candida* spp., followed by *S*. *cerevisiae*, *Y. lipolytica*, and *R. mucilaginosa*. The results of antimicrobial susceptibility testing revealed that all yeast isolates showed notable resistance to fluconazole and itraconazole.

**Conclusion:**

A significant correlation between antimicrobial resistance and biofilm formation was observed in several species. This study highlights the importance of the dangers of yeasts in food products and the extent of their impact on public health.

## Introduction

Meats and meat products contain essential components of the human diet, including protein, fatty acids, amino acids, minerals, vitamins, and other nutrients [1]. However, meat products can also be suitable media for microbial growth, which may affect the quality and safety of the products [2]. While bacteria and yeast are the main organisms responsible for meat spoilage, yeasts have a slow growth rate compared to bacteria and are commonly incapable of competing with bacteria for nutrients in refrigerated environments; however, the addition of antibacterial agents during meat processing hinders the growth of bacteria, giving fungi an opportunity to obtain the necessary nutrients for growth without competition [3].

Fungi can play useful or harmful roles in meat and other food products. While fungi can contribute to the improvement of desired properties in foods, their existence in food is generally considered a sign of food spoilage [4]. The production of lytic enzymes such as lipases and proteases is essential for the enhancement of flavor quality but can occasionally cause discoloration and poor flavors in meat products [5]. Some yeast species exhibit virulence traits that can increase their pathogenicity, such as the capacity to produce tissue-damaging hydrolytic enzymes and the ability to invade surfaces and form biofilms [6]. Biofilms are defined as cell communities that are adhered to a surface and encased in an extracellular matrix that is rich in self-produced polysaccharides. These biofilms can increase fungal resistance to many antibacterial [7, 8]. Although some fungi have intrinsic resistance to certain antifungals, unremitting exposure to antifungals can result in the development antifungal resistance, which may complicate treatment options. For example, bloodstream infections with resistant strains of *Candida* spp. can cause serious and even fatal health issues [9].

Several yeast species have been isolated from fermented meat products, including *Debaryomyces hansenii*, *Yarrowia lipolytica*, *Rhodotorula mucilaginosa*, and members of the *Candida* and *Cryptococcus* genera [4, 10]. These species cannot be differentiated on agar plates; therefore, advanced molecular identification methods are needed for species identification. The internal transcribed spacer (ITS) region is considered to be key to the identification of yeast species [11]. However, studies that investigate fungal microflora in frozen meat are scarce.

Therefore, this study aimed to screen frozen meat for the presence of yeasts and molds. In addition to evaluating biofilm formation, production of enzymes, and antifungal resistance, molecular characterization of the yeast isolates was performed.

## Materials and Methods

### Ethical approval

This study doesn’t require ethical approval because we purchased frozen meat samples from the market and not used live animals at any stage of the study.

### Study period and location

The study was conducted during September and October 2020 at Department of Microbiology, Faculty of Veterinary Medicine, South Valley University, Qena, Egypt.

### Sample collection

A total of 70 samples of locally produced frozen beef were purchased from 40 local stores in Qena City, Egypt. Individual samples were aseptically collected in a sterile plastic bag and immediately transported in an ice box to our laboratory for the analysis.

### Isolation of yeasts and molds

Each beef meat was aseptically sliced and 10 g of the sample was homogenized in 90 mL of 0.1% peptone water using a Stomacher Lab Blender (Stomacher^®^ 80 Biomaster Lab Blender, Seward, US). Samples were serially diluted and plated in duplicate on potato dextrose agar (PDA) (Oxoid, CM0139) plates (containing 200 g/L potato infusion, 20 g/L dextrose, 15 g/L agar, and 100 mg/L chloramphenicol). The plates were divided into two groups, and the first group was incubated at 37°C for 2 days to enumerate yeast, and the second group was incubated at 25°C for up to 5 days and until mold colonies were observed [12, 13]. In addition, the diluted homogenates were plated onto dichloran rose-bengal chloramphenicol (DRBC) agar plates (5 g/L peptone, 10 g/L glucose,1 g/L KH_2_PO_4_, 0.5 g/L MgSO_4_.7H_2_O, 25 mg/L dichloran (1 mL of 0.2% solution in ethanol), 0.025 g/L rose-bengal, 0.1 g/L chloramphenicol, and 15 g/L agar).

### Identification of fungal isolates

The morphological identification of fungal species was based on the macroscopic and microscopic characteristics. Purified yeast colonies were further identified at the species level using the VITEK 2 system and ID-YST cards according to the manufacturer’s instructions. Yeast suspensions were prepared by inoculating the colony with 3 mL of sterile saline (0.45% w/v) to match the turbidity of the 0.5 McFarland standard. Using an integrated VITEK 2 instrument, cards were automatically filled, sealed, and incubated at 35°C for 15 h. Yeast identification was obtained by comparing the profile results to the ID-YST database. (https://www.biomerieux-diagnostics.com/vitekr-2-2-yst-id-card).

### DNA extraction and polymerase chain reaction (PCR) analysis

Pure yeast cultures were inoculated into yeast peptone broth and incubated overnight at 28°C. Cells were then collected by centrifugation at 15,000× *g* for 3 min. Genomic DNA was extracted using a Yeast DNA Extraction Kit (Thermo Scientific, Cat. 78870, USA) according to the manufacturer’s protocol.

The PCR mixture consisted of 12.5 μL 2 × Master Mix RED (Amplicon, Denmark), 0.25 μM of each forward (ITS1: 5´-TCCGTAGGTGAACCTGCGG-3´) and reverse (ITS4: 5´-TCCTCCGCTTATTGATATGC-3´) universal fungal primers [14], 2 μL of extracted DNA, and sterile PCR water to bring the reaction mixture to a final volume of 25 μL. DNA samples were amplified in the thermal cycler as follows: 95°C for 5 min, 94°C for 45 s (35 cycles), 56°C for 45 s, 72°C for 1 min, and 72°C for 10 min [11]. Polymerase chain reaction products were electrophoresed on a 1.2% agarose gel in TBE buffer (Tris 90 mM, boric acid 90 mM, ethylenediaminetetraacetic acid 2 mM), stained with 0.5 g/mL ethidium bromide, and photographed under ultraviolet illumination.

### ITS-PCR sequencing and phylogenetic analysis

Purified PCR products were sequenced using an automated DNA Sequencer (ABI Prism 3730 Genetic Analyzer; Applied Biosystems, USA) with the ABI PRISM BigDye Terminator Cycle Sequencing Ready Reaction Kit (Applied Biosystems). The sequence alignment was performed using multiple alignment algorithms in MegAlign software (Windows version 3.12e, DNASTAR, https://www.dnastar.com/software/lasergene/megalign-pro/) and compared with similar sequences obtained from GenBank BLAST (https://blast.ncbi.nlm.nih.gov/blast.cgi). Phylogenetic analysis was performed for the yeast isolates using MEGA version 2.1 (https://www.megasoftware.net/) [15].

### Enzymatic activities

#### Screening for lipase activity

The lipase activity of yeast was performed on Tween 80 media, as previously described by Kumar *et al*. [16], with some modifications. In brief, yeast colonies were inoculated onto Tween 80 agar plates and incubated at ~27°C for 10 days. The zone of clearance surrounding the fungal colony was used as an indicator of the yeast’s capacity to degrade lipids.

Lipase activity assay

Lipase activity was spectrophotometrically determined using p-nitrophenyl butyrate (p-NPB) as a substrate [17, 18]. The unit (U) of lipase activity was defined as the amount of enzyme that liberated 1 μM of fatty acid per min at the defined conditions. The substrate solution was prepared by adding 0.1 mL of solution A (20.9 mg of p-NPB dissolved in 10 mL of absolute ethanol) to 10 mL of solution B (0.1 M sodium-potassium phosphate buffer containing 0.1 M NaCl, pH 7.2) in a volumetric flask to achieve a final concentration of 0.1 mM p-NPB. Aliquots of yeast cultures (20 μL) were added to 180 μL of the substrate solution and incubated at 37°C for 1 h. The release of *p*-nitrophenol was determined at 405 nm using a microplate reader at multiple intervals at 3, 7, and 14 days.

#### Screening for proteolytic activity

The ability of yeast isolates to produce proteases was determined using yeast carbon base (YCB) medium (pH 5.0; Oxoid) supplemented with 0.2% bovine serum albumin. Aliquots (10 μL) of the 48 h cultures (~10^6^ cells/mL) were spotted onto YCB plates and incubated at 37°C for 7 days. The formed zone of clearance around yeast colonies was then measured using a ruler [19].

Proteolytic activity assay

Protease activity was measured as follows: 2.5 mL of yeast culture (~10^8^ cells/mL) was added to an Erlenmeyer flask containing 100 mL of YCB medium supplemented with 0.01% sodium caseinate and 0.1% glucose. Flasks were then incubated with agitation (90× *g*) at 28°C for 3, 7, and 14 days and growth was determined from the optical density (OD) measurements at 600 nm [20].

### Biofilm production

Biofilm formation was evaluated using a 96-well microtiter plate assay, as previously described [21], with some modifications. First, one representative colony from each yeast isolate was added to 2 mL of brain heart infusion (BHI) broth and the broth was incubated for 24 h at 37°C. A BHI dilution (1:20) was added to the broth cultures, then aliquots (200 μL) of the cultures were placed into the wells of 96-well microtiter plates and incubated for 24 h at 37°C. After incubation, the microtiter plates were cleaned 3 times with distilled water and then inverted to dry. Aliquots (200 μL) of 1% crystal violet were poured into each well and the plates were incubated for 15 min. The plates were washed again (3 times with distilled water) and 200 μL of ethanol-acetone mixture (80:20 w/v) was added to different wells. A plate reader was used to measure the OD at 450 nm. The negative control was sterile BHI with no added yeast. The average OD values of the negative control were multiplied by the standard deviation to determine the cutoff (CO) value. Biofilm formation was classified based on the OD values into the following categories: Non-adherent/weak (OD ≤ 2 × CO), moderate (2 × CO < OD ≤ 4 × CO), and strong (4 × CO < OD) [22].

### Antifungal susceptibility testing

Antifungal susceptibility testing of yeast isolates was performed using the disk diffusion method [23]. In brief, yeast colonies were suspended in 5 mL of sterile saline to match the turbidity of a 0.5 McFarland standard. The yeast suspension was spread onto the surface of fresh Müller-Hinton agar (Oxoid) using a sterile cotton swab. The antimicrobials fluconazole (10 μg), itraconazole (10 μg), clotrimazole (10 μg), and miconazole (10 μg) were placed on the inoculated plates using a disk dispenser (Oxoid) and plates were incubated at 35°C for 24 h. The zones of inhibition were measured and interpreted as susceptible, intermediate, or resistant based on CLSI M44-A2 protocol breakpoints [24].

### Statistical analysis

Data were analyzed and graphics were generated using descriptive statistics SPSS version 28. Significance was considered at p < 0.05. A hierarchical cluster analysis was performed to group the yeast isolates based on their similarity and cluster aggregation.

## Results

**Figure-1:**
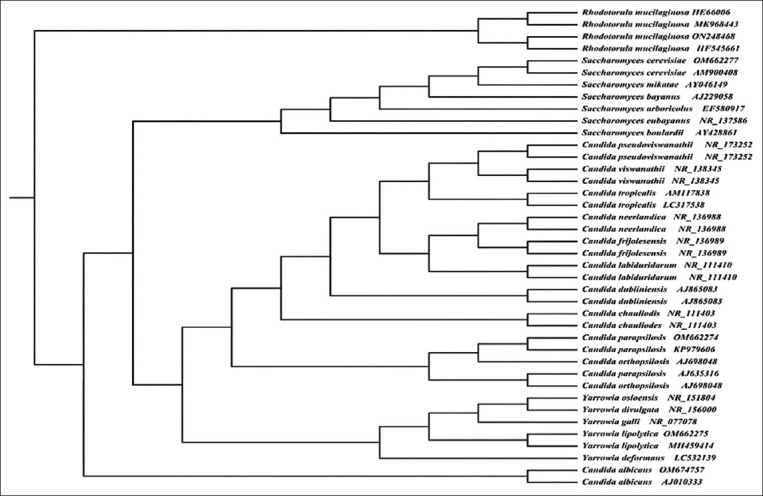
A neighbor-joining tree based on internal transcribed spacer sequences showing the phylogenetic relationships among yeast species that were isolated from frozen meat.

Out of the 70 meat samples tested, mold and yeast species were detected in 28 (40%) and 42 (60%) samples, respectively. Overall, the counts of fungal populations were significantly higher on DRBC than on PDA (R = 0.838 at p < 0.05) (Table-1). Our results showed that 30 (42.8%) samples were unacceptable, while 40 (57.2%) were acceptable. The identified mold species belonged to the genera *Aspergillus*, *Penicillium*, *Cladosporium*, and *Fusarium*. *Aspergillus* spp. were the most abundant species isolated from both media, followed by *Penicillium* spp., with occurrence frequencies of 34.5% and 28.5%, respectively (Table-1). Mold isolates were identified as *Aspergillus flavus*, *Aspergillus niger*, *Aspergillus fumigatus*, *Penicillium citrinum*, *Penicillium griseofulvum*, *Penicillium crustosum*, and *Cladosporium* spp. based on their colonial and microscopic characteristics (Table-1). Yeast isolates were identified by VITEK 2 as *Candida parapsilosis*, *Candida* *albicans*, *Y. lipolytica*, *Saccharomyces cerevisiae*, and *R. mucilaginosa*. The identities of these isolates were confirmed by ITS region sequencing and grouping with reference strains registered on a GenBank, with a similarity of 100% (Table-2). However, one isolate was unidentified by VITEK 2, but was later identified as *Y. lipolytica* using the ITS region sequence (Table-2). The phylogenetic relationships of the yeast isolates are shown in Figure-1. All yeast isolates belonged to two families, Dipodascaceae (*Yarrowia* and *Rhodotorula*) and Saccharomycetaceae (*Candida* and *Saccharomyces*).

**Table-1:** Counts (%) of fungal species isolated from frozen meat samples on DRBC and PDA agars.

Species	DRBC		PDA	
	
	TC (%)	F (%)	TC (%)	F (%)
*Aspergillus* spp.	750 (35.4)	24 (34.5)	530 (31.2)	22 (31.4)
*Aspergillus flavus*	320 (15.1)	9 (12.8)	200 (11.7)	7 (10.0)
*Aspergillus niger*	280 (13.2)	12 (17.1)	210 (12.3)	9 (12.8)
*Aspergillus fumigatus*	150 (7.1) 600 (28.3)	4 (5.7)	120 (7.01)	6 (8.5)
*Penicillium* spp.	100 (4.7)	20 (28.5)	350 (20.5)	19 (27.4)
*Penicillium citrinum*	280 (13.2)	5 (7.1)	80 (4.7)	4 (5.7)
*Penicillium griseofulvum*	220 (10.4)	8 (11.4)	140 (8.2)	7 (10.0)
*Penicillium crustosum*	75 (3.5)	7 (10)	130 (7.6)	9 (12.8)
*Cladosporium* spp.	35 (1.6)	15 (21.4)	50 (2.9)	10 (14.2)
*Cladosporium herbarum*	40 (1.8)	6 (8.5)	30 (1.7)	8 (11.4)
*Cladosporium cucumerinum*	40 (1.8)	9 (12.8)	20 (1.1)	2 (2.8)
*Fusarium oxysporum*	0 (0.0)	2 (2.8)	10 (0.58)	6 (8.5)
Mucor spp.	650 (30.7)	0 (0.0)	50 (2.9)	7 (10.0)
Yeast spp.	2115* (100)	7 (10.0)	710 (41.7)	10 (14.2)
Total count		--	1700* (100)	--

TC=Total count, F=Frequency among meat samples.

* Colony-forming units/g. DRBC=Dichloran rose-bengal chloramphenicol, PDA=Potato dextrose agar

**Table-2:** Identification of fungal isolates by ITS region and closest known species on GenBank.

Isolates	Identification by ITS region	Length of fragment	Accession No.	Closely related species		

				Species	Accession No.	Similarity (%)
1	*C. parapsilosis*	481	OM662274	*C. parapsilosis*	KP979606	100
2	*Y. lipolytica*	375	OM662275	*Y. lipolytica*	MH459414	100
4	*C. albicans*	480	OM674757	*C. albicans*	AJ010333	100
7	*S. cerevisiae*	750	OM662277	*S. cerevisiae*	AM900408	100
14	*R. mucilaginosa*	910	ON248468	*R. mucilaginosa*	HF545661	100

*C. parapsilosis=Candida parapsilosis, Y. lipolytica=Yarrowia lipolytica, C. albicans=Candida albicans, S. cerevisiae=Saccharomyces cerevisiae, R.mucilaginosa=Rhodotorula mucilaginosa*

The lipolytic and proteolytic activities of the yeast isolates are shown in Table-3 [25]. All yeast species were able to produce protease and lipase at different levels. The production of these enzymes increased after lengthy incubation (14 days) (Table-4). *C. parapsilosis* produced the highest level of lipase, followed by *C. albicans*, *Y. lipolytica*, *S. cerevisiae*, and *R. mucilaginosa*. The highest protease activity was observed in *C. albicans*, followed by *C. parapsilosis*, *S. cerevisiae*, *Y. lipolytica*, and *R. mucilaginosa*.

**Table-3:** Proteolytic and lipolytic activities of the isolated yeast species.

Yeast spp. (No. of isolates)	Lipolytic activity			Proteolytic activity		
	
	H*	M^#^	W^##^	H	M	W
*Candida parapsilosis* (10)	3*	6	1	1	7	2
*Candida* *albicans* (9)	2	5	2	4	3	2
*Yarrowia lipolytica* (2)	0	2	0	0	1	1
*Saccharomyces cerevisiae* (5)	0	3	2	0	3	2
*Rhodotorula mucilaginosa* (4)	0	2	2	0	1	3

* Enzyme activities were classified according to the zone diameters: high (>10 mm), moderate (3–10 mm), and weak (<3 mm) [25]. *H=High,

# M=Moderate,

## W=Weak

**Table-4:** Optical density of lipase and protease production by yeast species isolated from frozen beef meat.

Species (No. of isolates)	Lipase activity			Protease activity		
	
	3 days1	7 days	14 days	3 days	7 days	14 days
*Candida parapsilosis* (10)	0.47 ± 0.01	0.57 ± 0.01	0.65 ± 0.1	1.46 ± 0.01	1.65 ± 0.01	1.7 ± 0.26
*Yarrowia lipolytica* (2)	0.3 ± 0.01	0.4 ± 0.01	0.5 ± 0.09	1.11 ± 0.1	1.36 ± 0.13	1.46 ± 0.01
*Candida albicans* (9)	0.32 ± 0.03	0.4 ± 0.03	0.6 ± 0.12	1.67 ± 0.03	1.77 ± 0.03	1.83 ± 0.19
*Saccharomyces cerevisiae* (5)	0.14 ± 0.01	0.21 ± 0.01	0.47 ± 0.19	1.27 ± 0.03	1.48 ± 0.03	1.66 ± 0.07
*Rhodotorula mucilaginosa* (4)	0.05 ± 0.02	0.07 ± 0.02	0.43 ± 0.15	0.68 ± 0.35	0.95 ± 0.22	1.12 ± 0.3

^1^Incubation time (days). Values are means of triplicate determinations. OD measurements were performed at 405 nm and 600 nm for lipase and protease activities, respectively. OD=Optical density

The biofilm formation ability varied among different yeast species (Table-5). *Candida* isolates showed high biofilm formation abilities, with more than half of the isolates forming moderate-to-strong biofilms. However, only isolates of *C. parapsilosis* were able to form a strong biofilm.

**Table-5:** Biofilm formation of yeast species isolated from frozen beef meat.

Species (No. of isolates)	Strong biofilm	Moderate biofilm	Weak/no biofilm
*Candida parapsilosis* (10)	2*	2	6
*Yarrowia lipolytica* (2)	0	1	1
*Candida albicans* (9)	0	6	3
*Saccharomyces cerevisiae* (5)	0	3	2
*Rhodotorula mucilaginosa* (4)	0	1	3

Biofilm formation was classified based on the optical density (OD) values into the following: non-adherent/weak (OD≤2 × CO), moderate (2×CO<OD≤4 × CO), and strong (4×CO<OD).

* Number of isolates

**Figure-2:**
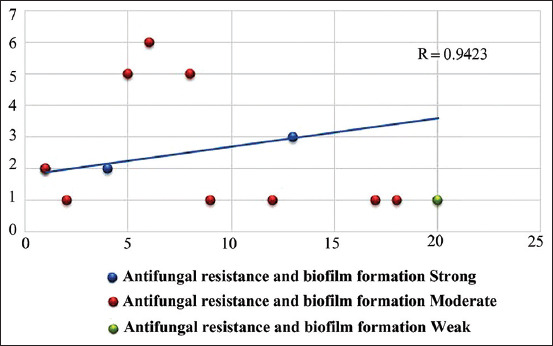
Relation between antifungal resistance and Biofilm formation. R = 0.942 showed a strong positive correlation between antifungal resistance and biofilm formation.

The antifungal resistance profiles of the yeast species isolated from frozen meat are shown in Table-6. Isolates of *Candida* spp. were resistant to fluconazole (57%), clotrimazole (73%), itraconazole (47%), and miconazole (36%). However, all isolates of *S. cerevisiae*, *Y. lipolytica*, and *R. mucilaginosa* were susceptible to miconazole. There was a significant correlation between antimicrobial resistance and biofilm formation in *Candida* spp. (R = 0.942; p < 0.05) (Figure-2).

**Table-6:** Antifungal resistance profiles of yeast species isolated from frozen meat.

Species (No. of isolates)	Antifungal agents			

	Fluconazole	Itraconazole	Miconazole	Clotrimazole
*Candida parapsilosis* (10)				
S	1*	1	5	1
I	4	3	5	2
R	5	6	0	7
*Candida albicans* (9)				
S	0	2	6	0
I	4	5	4	3
R	6	3	0	7
*Yarrowia lipolytica* (2)				
S	0	0	2	0
I	1	2	0	1
R	1	0	0	1
*Saccharomyces cerevisiae* (5)				
S	0	3	5	4
I	2	2	0	1
R	3	0	0	0
*Rhodotorula mucilaginosa* (4)				
S	3	2	4	3
I	0	1	0	0
R	1	1	0	1

S=Susceptible, I=Intermediate, R=Resistant.

* Number of isolates

## Discussion

Overall, the fungal species isolated from the frozen beef meat samples were diverse, with *Aspergillus* and *Penicillium* being the most dominant genera. Yeasts can contaminate meat at several points during the manufacturing process. Significantly higher counts of fungi on DRBC compared to on PDA (0.838 at p < 0.05) were observed in our results (Table-1). These results are supported by those of a previous study [26], which reported that dichloran was an important component in DRBC and may have been more effective in inhibiting the growth of *Mucor* and *Rhizopus* spp., so counting is easier, especially for small-sized colonies. On PDA media, *Mucor* and *Rhizopus* spp. grow more densely and cover a large number of colonies; therefore, the count is often unclear, and many colonies cannot be seen or counted on PDA; this explains the presence of increased numbers of molds on DRBC compared to PDA. In addition, the presence of rose-bengal in this medium increases the intensity of the pink color, which plays a role in defining colonies and makes the detection of colonies very easy [27].

*Aspergillus* spp. were the most abundant species isolated from meat samples, followed by *Penicillium* spp. (Table-1). These findings are similar to those of a previous report [28] that identified *Aspergillus* and *Penicillium* as the most common molds in retail meats from Alexandria, Egypt. Other studies have reported that *Aspergillus* species were the most common isolates from meat and environmental samples [29, 30]. The environmental fungi *Aspergillus* and *Penicillium* are frequently used in food fermentation. However, some mold species are linked to meat deterioration and can produce cancer-causing substances, such as aflatoxins [31]. It was outside the scope of our investigation to examine whether or not these mold isolates were capable of producing mycotoxins. In addition, several other mold species, such as *Cladosporium* spp., *Fusarium* spp., and *Mucor* spp., have been isolated from meat products [32].

The identification of yeast species was difficult due to progressive changes in taxonomy. Phenotypic and genetic methods for yeast identification are increasingly being developed [33]. In this study, ITS sequencing was used to overcome the issue of phenotypic misidentification and unidentified isolates using the VITEK II system (Table-2). This methodology is supported by a previous study [34], which confirmed the role of the ITS gene in mold identification. The phylogenetic analysis revealed that the yeast isolates were clustered with closely related species registered on a gene bank that were isolated from patient, environmental, and food samples (Figure-1), indicating the different sources potentially involved in meat contamination [35].

The species of yeast observed in our meat samples were similar to those reported in other studies [36, 37]. While yeasts are frequently found in meat products, some yeast species can proliferate while the meat is being stored and lead to spoilage [31]. *Candida parapsilosis*, *Y. lipolytica*, *S. cerevisiae*, and *R. mucilaginosa* have been isolated from meat products in previous studies [4, 10, 38].

One well-described virulence factor that aids in the development of invasive candidiasis is the formation of hydrolytic enzymes, including proteinases and lipases [39]. Microbially generated lipases may play a role in the initiation of inflammatory cascades, host cell attachment, and the digestion of fat for nutrient acquisition during infection [40]. A previous study has reported the effects of lipase- and protease-producing yeasts on the sensory characteristics of meat products [41].

In this study, the species *C. parapsilosis* and *C. albicans* showed the highest lipase and protease activities compared to other species (Table-3), which may be due to the YCB-BSA at an acidic pH, a recognized condition that induces the secretion of these enzymes in *Candida* spp. [42]. Similarly, some previous studies [43, 44] have reported higher lipase and protease activities in *Candida* spp. compared to other species. In addition, it was observed that the production of enzymes increased when the incubation period was increased up to 14 days (Table-4). These results corroborated the findings of two previous studies [45, 46]. Variations in enzyme activity between yeast species may be influenced by a number of factors, including temperature, pH, and incubation time, some of which may favor one species over another [47].

Several studies have supported the role of *Y. lipolytica and S. cerevisiae* in the production of enzymes. *Yarrowia lipolytica* produces a number of acids that enhance changes in the flavor and taste of meat products [48]. The yeast *S. cerevisiae* is one of the most significant species used as a food additive that is considered safe for consumption [8]. However, *S. cerevisiae* has recently been recognized as an emerging opportunistic pathogen [49]. Therefore, further identification of *S. cerevisiae* isolates at the strain level is needed to discover their pathogenic potential.

The production of tissue-damaging hydrolytic enzymes (e.g., proteases) and the formation of biofilms are considered virulence factors in yeasts, especially *Candida* species [50]. In this study, the biofilm formation abilities were found to vary among yeast species, and isolates belonging to *Candida* species had higher biofilm formation abilities compared to other species (Table-5). These results are reinforced by those of prior studies [32, 51]. A previous study [52] explained the ability of *Candida* to form biofilms in the following manner: Unique cell wall proteins known as adhesins, each of which permits adherence to particular substrates, were responsible for the creation of *Candida* biofilms. The production of various adhesins is closely regulated by a number of signaling cascades, including the Ras/cAMP/PKA and MAP kinase-dependent filamentous growth pathways. Together, these mechanisms cause adhesion in response to stress, nutritional deficiency, or host-produced small molecules. These defense systems enable swift adaptation to demanding surroundings [53].

*Candida albicans* and *C. parapsilosis* can cause invasive candidiasis in humans and some of their strains can be transmitted through contaminated food [54]. Biofilm formation in food processing facilities may lead to recurring contamination of meat products. Biofilms can enhance yeast survival by increasing their resistance to antimicrobials, genetic exchange, and the synthesis of secondary metabolites [55]. However, some yeast species, such as *S. cerevisiae*, produce beneficial biofilms that are important for food fermentation [8].

In this study, isolates of *Candida* spp. were found to be resistant to various drugs belonging to the azole family (Table-6) and the observed resistance levels were considerably higher than the previously reported levels for clinical and food isolates [56, 57]. The difference in the susceptibility of yeast to different antifungals may be linked to increased efflux pump activity, mutations in genes encoding drug target enzymes, and alterations in the composition of both the cell membrane and the cell wall [58]. In our study, the results revealed a significant correlation between antimicrobial resistance and biofilm formation (Figure-2). It is noteworthy that the *Candida* species with biofilm formation abilities also showed high resistance to azoles. These results are supported by the findings of Seneviratne *et al*. [59]. Perumal *et al*. [25]found that the resistance ability of biofilms to antifungal agents was highly comparative to many planktonic cells. Our results showed that 30 (42.8%) samples were unacceptable, while 40 (57.2%) were acceptable. Our results showed that 30 (42.8%) samples were unacceptable, while 40 (57.2%) were acceptable according to ICMSF [60] and Egyptian standards [61] which recommend that samples must be free from yeast and mold.

## Conclusion

Phenotypic and genotypic methods played an important role in the identification of fungal diversity in frozen beef samples. Many yeast species were able to produce lipase and proteases, form biofilms, and some species were resistant to major antifungal agents. Considering that these characteristics might indicate beneficial roles in some species and not in others, future studies are warranted to investigate the pathogenicity of these species and the effect of fungi on the sensory characteristics of meat products.

## Authors’ Contributions

WY and HMAM: Conceptualization and methodology. WY, HMAM, and RFM: Collection of the samples, laboratory work, and drafted the manuscript. SFA: Collection of samples, laboratory work, data analysis, and drafted the manuscript. WY, HMAM, and SFA: Manuscript revision. All authors have read, reviewed, and approved the final manuscript.
